# Dietary Perspectives and Practices during Pregnancy in Rural Amhara Region of Ethiopia: An Exploratory Qualitative Study

**DOI:** 10.1016/j.cdnut.2023.100079

**Published:** 2023-04-17

**Authors:** Firehiwot Workneh, Sitota Tsegaye, Hanna Amanuel, Michelle Eglovitch, Tigest Shifraw, Fisseha Shiferie, Amare W. Tadesse, Alemayehu Worku, Sheila Isanaka, Anne CC Lee, Yemane Berhane

**Affiliations:** 1 Department of Epidemiology and Biostatistics, Addis Continental Institute of Public Health, Addis Ababa, Ethiopia; 2 Department of Nutrition and Behavioral Sciences, Addis Continental Institute of Public Health, Addis Ababa, Ethiopia; 3 Department of Pediatric Newborn Medicine, Brigham and Women’s Hospital, Harvard Medical School, Boston, MA, United States; 4 Department of Reproductive Health and Population, Addis Continental Institute of Public Health, Addis Ababa, Ethiopia; 5 Infectious Disease Epidemiology, London School of Hygiene and Tropical Medicine, London, United Kingdom; 6 Departments of Nutrition and Global Health and Population, Harvard T.H. Chan School of Public Health, Boston, MA, United States

**Keywords:** dietary perspectives, dietary practices, pregnancy, fasting, food restriction, alcohol use during pregnancy, qualitative methods, Ethiopia

## Abstract

**Background:**

Nutrition during pregnancy has lifelong impacts on the mother and fetus. In Ethiopia, nearly a third of pregnant women experience undernutrition. When designing nutrition interventions during pregnancy, it is important to understand existing dietary perspectives and practices in local communities.

**Objectives:**

To explore the processes that shape dietary perspectives and practices during pregnancy in rural West Gojjam and South Gondar Zones of the Amhara region in Ethiopia.

**Methods:**

From October to November 2018, we conducted 40 in-depth interviews with pregnant women (*n* = 16), family members (*n* = 12), and healthcare providers (*n* = 12) using a semistructured interview guide. Interviews were conducted in Amharic, transcribed in Amharic, and translated into English. We used a thematic analysis approach to organize data per the predefined topic areas and identify emerging themes, as well as barriers and enablers to healthy nutrition during pregnancy.

**Results:**

Pregnant women and their family members recognized the benefits of a diversified diet to promote the health of the mother and the fetus. However, participants reported low dietary diversity because of limited access to nutritious foods and particular perspectives on food restrictions during pregnancy. The common practice of religious fasting also limited pregnant women’s dietary intake. Women reported restricting their food intake in later pregnancy because of loss of appetite, as well as concerns about having a large infant, which might complicate delivery. Intake of locally made alcoholic drinks (*Tella*) was reported among pregnant women because participants thought it had low levels of alcohol that would not harm the fetus.

**Conclusions:**

Although participants understood the importance of a healthy and diverse diet in pregnancy, we identified several barriers and perspectives regarding nutrition during pregnancy. Low income and lack of access to diverse foods, particularly in certain seasons, religious fasting, intentional food restrictions to limit the size of the infant, and alcohol use were commonly reported. Locally appropriate counseling and interventions should be developed, with an emphasis on increasing access to and consumption of diverse foods. *Curr Dev Nutr* 2023;x:xx.

## Introduction

Nutrition during pregnancy affects infant, child, and maternal health [1]. All stages of the life cycle require proper nutrition and a healthy diet; however, nutritional needs are highest among pregnant and nursing mothers because of the increased metabolic demand for the majority of nutrients [2]. Maternal undernutrition is defined as having a BMI (in kg/m^2^) of <18.5 or mid-upper arm circumference <23 cm. In 2021, it was estimated that 1 in 3 pregnant women in Ethiopia experienced undernutrition and that the prevalence was higher among those residing in rural areas compared with the urban [3]; ∼53% of pregnant women in Ethiopia experience inadequate dietary diversity that is defined as the number of food groups or items consumed over a reference period [4].

Key causes of undernutrition in pregnant and breastfeeding women include poverty, food insecurity, limited access to nutrient-rich foods, low nutritional quality of diets, and low nutritional intake [[5], [6], [7], [8], [9]]. In addition, inappropriate food preparation and storage, unequal food distribution within households, dietary taboos, and frequently occurring infectious diseases often exacerbate the burden and effects of undernutrition in low and middle-income countries, particularly in sub-Saharan Africa [5,[7], [8], [9]]. Strategies at the international level designed by the WHO and national levels designed by the Ministry of Health emphasize a healthy diet for all with an emphasis for pregnant women. However, in low and middle-income countries, a significant proportion of pregnant women consume inadequate and nutrient-poor diets [[10], [11], [12]]. Maternal undernutrition is associated with adverse pregnancy outcomes, including low birth weight, preterm birth, and small for gestational age [2,[13], [14], [15]], whereas improved nutrition in pregnancy is associated with improved maternal immunity, pregnancy and birth outcomes, and mother and child health and cognitive development [11,16].

However, the local environment, customs, and dietary behaviors during pregnancy may affect the diet of pregnant women as well as the availability and access to diverse nutrient-rich foods. Understanding local dietary perspectives and practices during pregnancy is crucial to the design and implementation of nutritional interventions. In light of this, the objective of our study was to qualitatively study the dietary perspectives and practices of pregnant women, as well as the perspectives of family members, health center staff, and health extension workers (HEWs) in rural communities in West Gojjam and South Gondar Zones of the Amhara region in Ethiopia. We also sought to gain perspectives on nutritional advice and counseling for pregnant women from their family members and local healthcare providers (HCPs).

## Methods

This formative qualitative study was conducted from October to November 2018 to inform the design of interventions for the “Enhancing Antenatal Nutrition and Infection Treatment for Maternal and Child Health” study (https://www.isrctn.com/ISRCTN15116516) [17]. The overall aim of the main study was to determine the impact of a combined package of antenatal care (ANC) interventions to enhance maternal nutritional status and manage maternal pregnancy infections on newborn size at birth [17]. This qualitative research was performed before the intervention study to inform the design of the nutrition intervention package and the nutrition counseling.

### Study site

The site selected was a potential site for the Enhanced Nutrition and Antenatal Infection Treatment study. It was conducted in 4 rural health centers in West Gojjam and South Gondar Zones, Amhara Ethiopia, where there were high rates of maternal undernutrition, recurrent drought, and food insecurity [5,18]. Food insecurity is one of the major predisposing factors of maternal undernutrition. The 2011 Household Consumption & Expenditure survey found that 42.5% of households in the Amhara region were food insecure, which is higher than the 33.6% national average [19]. Unstable weather conditions and recurrent drought influence food security in the Amhara region and Ethiopia more broadly. In addition, traditional farming practices such as monocropping are associated with the monotonous consumption of micronutrient-deficient crops. The study areas depend highly on subsistence farming and often harvest crops once per year, which limits diet diversification in the area.

### Study design, participants, and sampling procedures

We conducted this qualitative study by interviewing pregnant women, their partners, family members, and community-based HCPs. Pregnant women were interviewed to hear first-person accounts of their experiences and their suggestions. Their partners and family members were interviewed because families play a central role in nutritional practices and decision making. Finally, HCPs were interviewed because they are key stakeholders in nutrition interventions and support pregnant women through ANC visits at the health centers, in the monthly maternal health conference at health posts, and during home-to-home visits.

### Participant recruitment

One health center was selected from each of the 4 woredas (districts) identified for the main study. We selected health centers with high ANC volume based on the annual performance report obtained from the regional health bureau for the year 2017/2018 (2010 Ethiopian Calendar). We used a purposive sampling procedure to identify study participants with varying parity. Husbands or other relatives accompanying the mother at the ANC were identified, some of whom were also religious leaders and community administrators. Knowledge about the culture and having real experience with pregnant women were used to identify community members. HCPs with >2 y of working experience in the study area were selected because they were perceived to be aware of community perspectives. We received verbal and written informed consent for all study participants. Participants without literacy in Amharic had a consent form explained to them by a family member (if in attendance) or study staff and would verbally consent to the study. For health workers, eligibility criteria included current provision of maternal and child health services and serving for 2 or more y in the surrounding communities of the selected health center. We based our sample size upon the principle of saturation. On the basis of our prior experience in this study area and population, we anticipated a sample size of 10–15 per group required to reach both code and meaning saturation [20] and to enable a rich understanding of key issues and themes. A total of 16 pregnant women, 12 family members and partners, and 12 HCPs were included in the study (Supplemental Figure 1).

### Data collection

Data were collected through in-depth interviews (IDIs) using a semistructured interview guide. Drawing on existing literature, the interview guide was developed by the study team to capture the main themes related to dietary perspectives and practices in pregnancy, including food preferences, preparation decisions, consumption frequency, fasting, dietary restrictions, family sharing practices, and alcohol consumption. HCPs were interviewed about nutritional counseling during ANC visits to understand their perspectives and the existing landscape of nutrition counseling. The interview guides were developed in English and then translated to Amharic, the local language of the study area. All interviews were conducted face to face by trained research assistants (RAs). RAs had prior training in public health or social sciences, as well as relevant experience in qualitative data collection techniques, including IDIs. They also received training on the study objective, the specific data collection procedures, and on research ethics. RAs were paired when conducting interviews. The Ethiopian researchers supervised the field data collection and conducted daily debriefing sessions. All IDIs took place on the premises of the health center when the mothers came for the ANC follow-up. The interview was conducted in a separate place to keep the privacy of the participants.

Interviewers gave participants a detailed explanation of the study objectives to obtain consent for conducting the IDI. HCPs at the 4 health centers were interviewed at a convenient time when the patient load was lighter. With the participant’s permission, the interviews were recorded using digital audio recorders along with interview notes. Pregnant women were interviewed for an hour on average, whereas family members and HCPs were interviewed for an average of 30 and 45 min, respectively.

### Data analysis

The RAs verbatim transcribed and translated the interviews into English for analysis. We used thematic analysis within predefined topic areas [21]. Three authors reviewed the full-text translations of all interviews and assigned interview content to the major predefined topics. We coded the interview transcripts line by line according to the predefined topics of dietary practices, food restrictions, and fasting. New codes were assigned for issues outside the predefined topics and later grouped into thematic areas. We used Excel to group themes within our larger topic areas. We then integrated the new themes and their corresponding interview excerpts with the associated field notes. Illustrative quotations were integrated with the narrative description of the study findings.

### Ethical considerations

The study was approved by the institutional review boards (IRBs) of Addis Continental Institute of Public Health (Addis Ababa, Ethiopia) (/IRB/007/2018) and the Massachusetts General Brigham IRB (Boston, Massachusetts, United States) (2018P002479). Support letters were obtained from Amhara Public Health Institute (Bahir Dar, Amhara region, Ethiopia), and permission was obtained from the Zonal Health Department and health centers. Informed written consent after receiving detailed information on the purpose of the study was obtained from all the participants in the 3 groups (pregnant women, community members, and HCPs). The participants were informed that their responses and recordings would be kept anonymous, and reports/publications emerging from the study would not include individual identifiers. Data were anonymized at the point of interview and no names were recorded and unique study IDs were assigned for each participant. Participants were not compensated and did not directly benefit from study participation. Subjects were not directly informed of the results of the study but the findings were presented at each of the study health centers.

## Results

### Participant characteristics

The interviewed pregnant women (*n* = 16) were between the ages of 18 and 36 y (Table 1). Four (25%) women were primiparous. Ten of the pregnant women were in the 3rd trimester, 4 in the 2nd, and 2 were in the 1st trimester. Family members (*n* = 12) were all men: 8 were husbands, 2 fathers, and 2 other relatives who accompanied their family members to the ANC visit. The HCPs consisted of 8 midwives and 4 HEWs with >5 y of work experience within their communities.

**TABLE 1:** Sociodemographic characteristics of study participants, *n =* 40

Participant groups	Pregnant women	Family members	Healthcare providers
*n*	16	12	12
Age (average)	26	41	27
Description			
	1st trimester *n =* 2 (13%)	Husband *n =* 8 (67%)	Midwifes/nurses *n =* 8 (67%)
	2nd trimester *n =* 4 (25%)	Father *n =* 2 (17%)	Health extension workers *n =* 4 (33%)
	3rd trimester *n =* 10 (63%)	Other relative *n =* 2 (17%)	
Education level			
No formal education	7 (44%)		
Some primary or secondary	9 (56%)	6 (50%)	
Degree			4 (33%)
Diploma			3 (25%)
Level 4			5 (42%)

The result presentation begins with findings on the dietary context. We then describe processes facilitating and hindering access to nutritious and diverse diets during pregnancy.

### Dietary context

Most participants indicated that cereals that frequently came from their farms are consumed at home. The staple food as reported by pregnant mothers and community members is Injera (flatbread made of either teff, millet, maize or mixed grains), which is typically eaten with stew “Shiro,” made from a powder of pea, grass pea, or chickpeas. In the study area, stews made of seasonal vegetables are less-frequently served. The long fasting seasons influence the availability of poultry and dairy in the market as revealed by participants. Because of the high prevalence of fasting, meat availability is usually reserved to market days (certain days where people come from different area to buy and sell groceries and other items, usually once or twice a week based on the area), and beef is usually consumed on occasions such as a wedding or holidays. Depending on the season, participants mentioned that they consume freshly harvested grains such as fresh beans, millets, maize, barley, or chickpeas as snacks in between meals. Usually, roasted barley, chickpea, sorghum, or other grains are served when having coffee. With seasonal variations in production, respondents mentioned limited availability of fruits, vegetables, and grains. In addition, access is dependent on the economic status of the household (Table 2).

**TABLE 2:** Available food items in the local market mentioned by the study participants

Food categories	Food items available in the study catchment (South Gondar and West Gojjam, Amhara Ethiopia)
Fruits	Banana
	Orange
	Papaya
	Mango
Vegetables	Cabbage
	Kale
	Potato
	Tomato
	Carrot
	Onion
Poultry and meat	Chicken
	Meat (goat, beef, and lamb)
Dairy	Milk
	Butter
Staple crops	Teff
	Maize
	Barley
	Millet

### Facilitators of healthy dietary practices: community awareness and prenatal counseling

The interview participants emphasized the importance of increasing food consumption and diversifying the diet during pregnancy. According to the participants, monotony and inadequate consumption of food might have a detrimental impact on an infant’s health. One pregnant woman said: “During pregnancy, healthy foods to consume include eggs, vegetables, fruit, meat, and milk.” A family member said: “Pregnant women should consume a variety of meals, such as meat, eggs, vegetables, and fruits … banana and mango, as well.” In addition, participants discussed the importance of locally prepared liquid and solid porridges made from maize, barley, and millet, particularly for the postpartum period.

In addition to pregnant participants’ awareness of the role of diet in fetal growth, they expressed the importance of its connection to postpartum blood pressure, hemorrhage after delivery, and child cognitive development. One pregnant woman noted: “Nutritious food consumption throughout pregnancy is crucial for the development of a healthy baby, as well as for the mother’s strength and health during delivery, and the smooth completion of the pregnancy.”

Family members also noted the benefits of having a balanced diet during pregnancy. One family member expressed: “Proper diet is very essential during pregnancy for the mother to gain weight and prevent anemia … it enables her to have more blood (a local description for not having anemia). Additionally, it will help the fetus to gain adequate weight.” Another family member noted: “It is important to have nutritious food as it helps the mother to have a healthy baby, to have complete organs and also to prevent any disability.”

Pregnant women receive counseling during the regular antenatal visits to the health facilities as indicated by the HCPs. The counseling focuses on increasing food consumption during pregnancy, diversification, and improved adherence to iron folate. HCPs mentioned that the intensity of the counseling increases for mothers with inadequate weight gain in consecutive ANC contacts or who are found to be malnourished during the ANC visits. One midwife explained: “We counsel pregnant mothers on a variety of nutrition related topics such as the need to maintain a balanced diet, the need to diversify and also to increase intake by having in a snack or one additional meal during the ANC visits.” In addition, as part of the health education program, the local HEWs have worked to change the customs of avoidance and fasting, and try to persuade pregnant women to eat when they are hungry. The monthly pregnant women conferences that are held at health posts were used as a platform for providing health education related to pregnancy. HEWs provide a range of nutrition counseling including demonstrating how to make diversified meals and pregnant women shared experiences with peers and HCPs. The pregnant women also noted that they learned about nutrition during pregnancy from HCPs at a nearby health facility during their antenatal visits.

### Barriers to healthy diet: food insecurity, food restrictions, and alcohol consumption

Despite the awareness of dietary requirements in pregnancy, many pregnant women expressed that, because of inadequate household income, they could not afford to buy fruits and other food types from the local market. The availability and cost of food items varied greatly between the dry and rainy seasons. Some participants expressed that, because of these financial constraints, pregnant women often eat the same diet as other partners and family members. One family member said: “… no one is concerned about the diet of the pregnant woman that much. They eat the same foods as the other members of the household, both in terms of quantity and frequency.” In addition, although eggs and other dairy products, such cheese and butter, were more readily available at home, women had to frequently sell them in exchange for money for other essentials.

Within the family-wide context of inability to afford food, mothers often experienced the greatest constraints. Participants noted that in certain households, the husband had priority for access to food. Only after the husband had completed his meal, would wives be able to eat with their children. As one pregnant woman said: “If my spouse decides not to eat his lunch, I’ll likewise skip lunch and join him for supper instead. If he didn’t have a meal, I feel like I shouldn’t either.” HEWs also mentioned that it is customary for wives to wait for their husbands to arrive before eating, even though they may be hungry. In addition, as one family member noted, “even pregnant women should share their snacks, and it is not accepted by the community not to share.” A pregnant woman similarly stated: “I share my snacks with family members or a guest who comes to my house while I am eating, I will give them separately if I have more food but if that all there is, I will share what I have served for myself. Otherwise, they will think that I am being stingy.”

Other times, women may miss meals entirely because they are too busy with household chores and farm work; and they often consume anything between meals.

#### Food restrictions

Various restrictions around the preparation of food and type of foods were reported. Women and family members reported that pregnant women should consume cooked dishes, but not at high temperatures or right off the oven because they believed that this may cause pain and gastrointestinal irritation. Moreover, consumption of cold food was reported to be culturally restricted. A pregnant woman shared: “I don’t want to eat cold food since it can make me ill and also cold foods reduce appetite.” Another pregnant woman noted: “as much as possible, pregnant women should eat fresh meals at a medium temperature to prevent typhoid infection. Additionally, eating foods with high temperature while pregnant may lead to hair loss to the newborn baby.” Lastly, a family member noted: “Cold food is vulnerable to viruses, particularly food borne illnesses, but if the food is hot and allowed to cool down gradually, it will be fresh and safe for her to consume.”

Fasting is a common religious practice that is highly supported by religious authorities, elders, neighbors, and other family members. The Orthodox Tewahedo Church is the dominant church in the study area and all of our participants identified themselves as Orthodox. According to the church doctrine, >200 days per year are considered fasting days, and no animal products including meat or dairy products are allowed. The fasting includes skipping meals and water until 3 pm, until the daily church prayer ends during fasting seasons. Like any other followers of the Orthodox Tewahedo Church, pregnant women are expected and encouraged to fast. Even though it is discouraged by some, pregnant participants emphasized that fasting is necessary and that every woman must do it throughout pregnancy. During the religious fasting seasons, eating foods from animal source foods is to be avoided as well as skipping meals mainly breakfast. Although consuming anything, including water, is not permitted until 3 pm (when church prayer concludes), pregnant women typically fast until noon. Despite the recommendation of religious leaders to not fast, pregnant women feel that they must fast because it is a spiritual practice, and therefore will not harm the fetus. As one pregnant woman noted: “even if no one encourages me, I must fast. It would be irreligious to eat and might make God to be angry at me.” Another pregnant woman stated: “How am I able to consider not fasting? I don’t intend to stop fasting since it is spiritual and something that God commands.” Lastly, a family member said: “A woman should fast even if she is pregnant unless she has a specific health issue that prevents her. Even I, her husband or a spiritual leader, won’t let her break her fast.”

On the contrary, fasting is not a common practice during the postpartum period. It is believed that mothers need to be physically fit and healthy to breastfeed and raise their children. To nurse and nurture their infants, mothers consume proteins and fats right after birth. Although HEWs and healthcare professionals provide information about maternal nutrition, they expressed that many pregnant women do not eat the recommended foods when fasting.

Participants also spoke about a community-wide perception that some food groups pose a risk, such as *nifro* (boiled wheat or chickpea), and hot drinks. Consumption of such foods were thought to cause abdominal cramps, heartburn, or stomach aches for the mother and also have an impact on the fetus’s health.

Loss of appetite, feeling of fullness, and abdominal discomfort were mentioned as reasons for decreasing food consumption during pregnancy. Participants also shared community perceptions that eating more frequently would cause the fetus to gain weight and complicate the delivery. As a result of this misperception, many pregnant mothers minimize food intake during the 3rd trimester to reduce delivery complications. As a family member stated: “It is common for mothers to limit the amount of food they eat while pregnant compared to a non-pregnant state.” Another family member similarly noted: “A pregnant mother should drastically restrict her food intake in the latter stages of her pregnancy since the baby became big and she may experience obstruction during labor.”

#### Alcohol consumption

Although water was mentioned as the preferred beverage during pregnancy, some mothers stated that they preferred to drink *Tella* (a home brewed alcoholic beverage). Almost all participants agreed that alcohol intake should be limited when pregnant, although family members encouraged women to consume *Tella* because it was thought to have a low alcohol concentration. Pregnant women and family members expressed that drinks with high alcohol contents such as *Arekie* (home distilled liquor) should be avoided during pregnancy. One family member said: “Mothers during pregnancy should not get alcohol but it is okay if she takes a small amount of *Tella*.” Another family member referred to it as a “soft drink” noting: “they [pregnant women] can drink soft drinks including the local beverage called *Tella*.”

## Discussion

This study showed that despite wide-spread awareness about the importance of nutrition during pregnancy, the diet in the study area was characterized by food insecurity, restrictive dietary perspectives and practices, and permissibility of a local alcohol drink.

Lack of access to nutritious foods limited dietary intake and dietary diversity in this population. It is widely recognized that an increase in nutrients and food diversification lowers risk of micronutrient deficiency during pregnancy [5]. However, access to and consumption of fruits and vegetables even from the local markets is influenced by the household income level [5,[22], [23], [24]]. Skipping meals and “being served last” contribute to both macro- and micronutrient deficiencies for pregnant women [25].

Dietary perspectives and practices were also shaped by restrictions because of religious fasting and dietary taboos. In Ethiopia, dietary diversity is generally limited by low consumption of animal source foods, fruits, and vegetables [[26], [27], [28], [29]]. In the Amhara region, consumption is further restricted due to cultural and religious practices such as the absence of snacks between meals, heavy workloads forcing people to skip meals, and prolonged religious fasting seasons [[30], [31], [32]]. Participants emphasized that pregnant women are encouraged to fast unless they have health issues. Despite church leaders’ encouragement of the consumption of nutrient-rich meals, without any restriction, participants noted that it is considered “irreligious” to not fast. Foods consumed during fasting seasons tend to be less nutrient dense than those eaten during the nonfasting seasons [25]. Fasting restriction results in suboptimal consumption of micronutrients and lower total macronutrient/calorie intake. This contributes to maternal undernutrition and may have long-term effects on the health of both the mother and developing fetus [24,25,33]. In addition to the restriction of certain types of foods, the temperature of meals was also a factor in shaping what pregnant women were recommended to eat. Restrictions on the types of food during pregnancy may limit the intake of certain nutrient-rich diets, making pregnant women more susceptible to micronutrient deficiencies [34] and low dietary diversity [22].

Finally, in line with previous studies, this study revealed a common misperception that consumption of *Tella*, locally brewed alcohol, during pregnancy is appropriate. *Tella*, a traditional fermented alcoholic drink, resembles a beer with an estimated alcohol content between 2% and 6% depending on the fermentation and raw material used [35]. Previous studies have shown that alcohol consumption during pregnancy increases risk of adverse birth outcomes, particularly preterm delivery and low birth weight [32,36]. Fetal alcohol syndrome is a consequence of prenatal alcohol consumption that leads to neurocognitive deficits, growth deficiency, and facial dysmorphology in the unborn child [37,38]. The use of alcoholic drinks is consistently related to high rates of fetal alcohol syndrome, which is estimated to be 7.9% in Ethiopia [39]. Information on the effects of alcohol on maternal and fetal health, as well as support to minimize harm from the use of alcoholic drinks, is important in this region.

### Implications for intervention design

This study was conducted to inform the design of a maternal nutrition program for an intervention study [17]. Several key considerations for intervention designed emerged from these data. First, it was clear that educational initiatives alone are insufficient, because despite participants’ awareness of dietary needs during pregnancy, they have limited access to essential foods. Thus, nutrition interventions must move beyond educational campaigns and work to help families access needed foods or supplements through infrastructure (for example, supporting local markets), health systems or community-level support. Second, dietary practices are not an individual, but rather a socially embedded process in families (with sharing of food) and wider communities. It was clear that the religious practices and beliefs were critical in informing dietary practices for all, including pregnant women. Nutrition interventions should not only engage pregnant women, but should also engage partners, family members, and religious and other community members. Although the study’s implications are primarily systemic, this study did also inform aspects of the selection of a balanced energy protein supplement for the parent study: the selection of a vegan product that could be consumed during fasting periods and accounting for the sharing of portions with family members in the amount of nutrition supplement provided.

### Strengths and limitations of the study

The qualitative approach of this study allowed for a greater depth of inquiry not only on dietary practices during pregnancy, but also on the rationales for such practices. It allowed for deeper exploration of differing perspectives by triangulating findings from family members, HCPs, and pregnant women themselves. It also gave us insight into local efforts, at the scale of families and communities, to improve perinatal nutrition. We interviewed midwives and HEWs because they engage most deeply with pregnant women and their families. However, social desirability bias was unavoidable.

In conclusion, low household income, food insecurity, restricted food practices because of fasting, and dietary taboos contributed to inadequate nutrient intake and lack of dietary diversity during pregnancy in rural Amhara region of Ethiopia. Formative research should be an integral component of designing nutritional interventions because contexts are likely to have unique cultural and structural issues that influence acceptability and adherence to interventions.

## Funding

This study was funded by the Bill and Melinda Gates Foundation (OPP1184363). Dr. Lee was supported by NICHD K23 HD091390*.*

## Author disclosures

FW, ST, HA, ME, TS, FS, AWT, AW, SI, ACCL, and YB, no conflicts of interest.

## Authors Contributions

The authors’ responsibilities were as follows—FW: participated in study guide development, field supervision, data analyses, interpretation of results, and wrote the manuscript; ST and HA: participated in manuscript writing; TS: participated in study guide development, field team training, and critical review of the manuscript; FS: participated in field supervision and reviewed the manuscript; HA, ME, SI, AWT, and AW: provided input during study guide development, analysis, and critical review of the manuscript; ACCL and YB: conceived the research questions, guided study tool development, analysis and interpreting results, and critical review of the manuscript; and all authors: read and approved the final manuscript.

## Data availability

Data described in the manuscript, code book, and analytic code will be available upon request pending application and approval.

## Appendix A Supplementary data

Multimedia component1 Multimedia component1
