# Supplementary material for: Dietary Perspectives and Practices during Pregnancy in Rural Amhara Region of Ethiopia: An Exploratory Qualitative Study
Source: Curr Dev Nutr. 2023 Apr 17;7(6):100079. doi: 10.1016/j.cdnut.2023.100079 (PMC10209480; doi:10.1016/j.cdnut.2023.100079)
Supplement: Multimedia component1 [file mmc1.pdf]

Dietary perspectives and practices during pregnancy in rural Amhara region of Ethiopia: an exploratory qualitative study

Firehiwot Workneh Abate

Addis Continental Institute of Public Health, Addis Ababa, Ethiopia

Email: firehiwotworkneh@addiscontinental.edu.et OR firehiwotwaciph@gmail.com

Participant recruitment flow chart

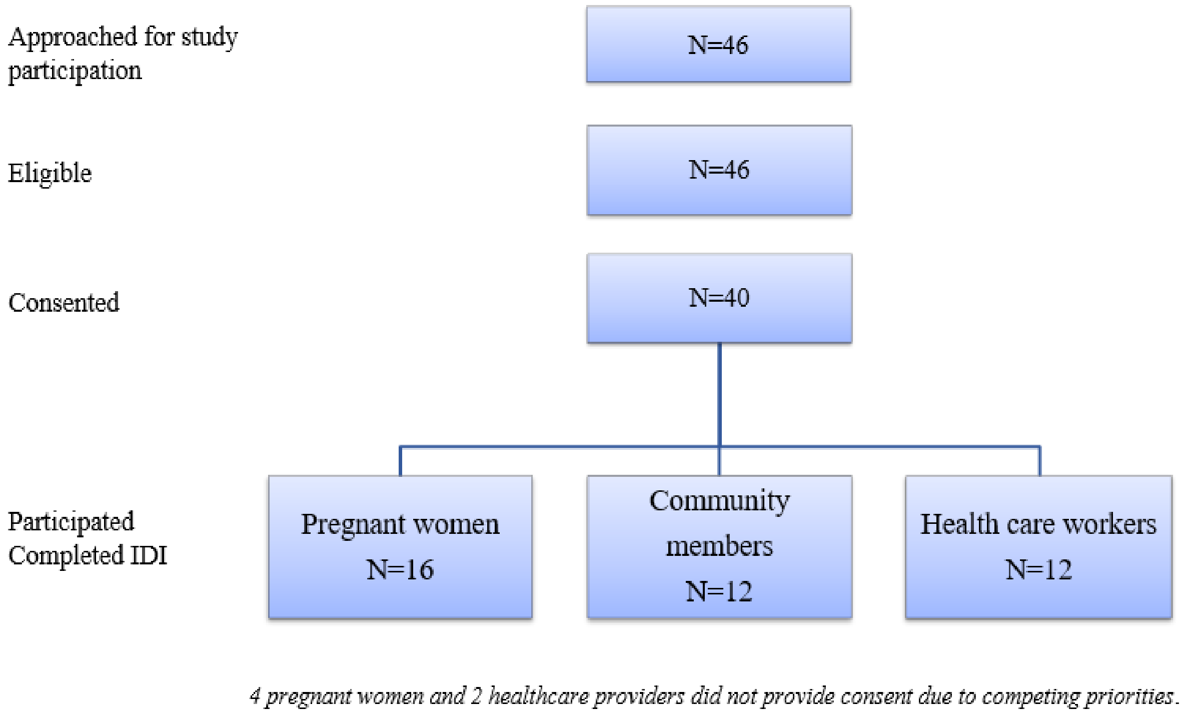

Supplemental figure 1: Participant recruitment flow chart, n=40.
